# Analysis of Academic Literature on Environmental Valuation

**DOI:** 10.3390/ijerph17072386

**Published:** 2020-03-31

**Authors:** Francisco Guijarro, Prodromos Tsinaslanidis

**Affiliations:** 1 Research Institute for Pure and Applied Mathematics, Universitat Politècnica de València, 46022 Valencia, Spain; 2 Department of Economics, University of Western Macedonia, 52100 Kastoria, Greece

**Keywords:** environmental valuation, literature survey, willingness to pay, choice experiment, contingent valuation

## Abstract

Environmental valuation refers to a variety of techniques to assign monetary values to environmental impacts, especially non-market impacts. It has experienced a steady growth in the number of publications on the subject in the last 30 years. We performed a search for papers containing the term “environmental valuation” in the title, abstract, or keywords. The search was conducted with an online literature search engine of the Web of Science (WoS) electronic databases. A search of this database revealed that the term “environmental valuation” appeared for the first time in 1987. Since then a large number of studies have been published, including significant breakthroughs in theory and applications. In the present work 661 publications were selected for a review of the literature on environmental valuation over the period 1987–2019. This paper analyzes the evolution of the leading methodologies and authors, highlights the preference for the choice experiment method over the contingent valuation method, and shows that relatively few papers have had a strong impact on the researchers in this area.

## 1. Introduction

Environmental valuation has traditionally been considered in the context of non-market valuation. Its aim is to obtain a monetary measure of the benefit or cost to the welfare of individuals and social groups of environmental improvement interventions or the consequences of environmental degradation [1,2]. However, the ultimate goal is not to value a (non-market) environmental good in monetary terms, but to provide decision-makers with the necessary tools to take the appropriate political initiatives to efficiently allocate resources, impose taxes and design compensation schemes [3,4], even after assuming the difficulties of developing theoretically grounded practical policy tools and avoiding political manipulation [5].

Environmental valuation methods have been used to determine the benefits and costs related to the use of environmental goods, improving their conditions or remedying environmental damage and must consider the complexity of the area. For example, the economic benefits of national parks extend beyond tourism; natural amenities and recreation facilities often serve to attract and retain people, entrepreneurs, businesses, and retirees [6]. On the other hand, some researchers have provided evidence of how worsening environmental conditions can affect the value of other goods. For example, noise and air pollution from road traffic have been reported to negatively impact real estate prices [7,8], and [9] reported that 55% of those surveyed in Brisbane (Australia) considered that noise adversely affected the value of their property.

Economists have traditionally developed tools to measure environmental values by estimating individuals’ willingness to pay to benefit from environmental goods. The costs associated with environmental deterioration are measured by the loss suffered by the individuals who benefited from the damaged good, and deciding the appropriate compensation for losing the benefit (willingness to accept) [10,11].

**Figure 1:**
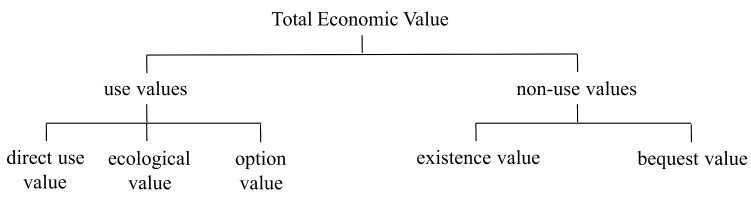
The concept of Total Economic Value of environment, taken from [12].

The general approach of Total Economic Value (TEV) combines all the different values, which are grouped according to the service provided by the environmental good (Figure 1). The use values are those derived from the actual use of the resource, while the non-use values are not related to its present use. The former includes the direct use value—the value derived from the direct use and exploitation of the environmental good, the ecological value—defined by the benefits that environmental goods provide to support forms of life and biodiversity and the option value—related to future use opportunities of the good. Non-use values are composed of the existence value—the value that individuals give to environmental goods for their mere existence—and the bequest value—the value estimated by individuals when considering the use of goods in the future by their heirs.

The aim of environmental valuation methods is to measure the values included in TEV. Although some authors have classified valuation methods from a more general perspective [13], the methods specifically related to environmental valuation can be classified as follows:

- Stated preference methods. These rely on hypothetical questions and estimate values by asking individual survey questions related to their preferences.
  - Contingent valuation method. Values are estimated in a hypothetical market based on surveys in which respondents are asked how much they are willing to pay for the use and conservation of an environmental good. The purpose of contingent valuation is to estimate individual willingness to pay for changes in the quantity or quality of environmental goods or services [3].
  - Choice experiment method. This method provides the respondents with alternative choices in which different environmental goods are defined by their attributes. According to [14], “the most significant advance in environmental valuation may be to move away from a focus on value and focus instead on choice behaviour and data that generate information on choices.”.
- Revealed preference methods. Environmental values are estimated by observing the values of market goods related to the non-market environmental good, such as the purchase of a home or visits to a recreational site.
  - Travel cost method. Values are estimated by accounting for the cost incurred by people who travel to visit an environmental good. The method assumes that the willingness to pay must be at least as large as the travel cost incurred.
  - Hedonic price method. Values are computed from the prices of traded goods. This approach is frequently used when the price of traded goods is influenced by environmental factors [8].

The field of environmental valuation has recently expanded both from a theoretical and practical point of view [15]. This paper aims to outline the advances made by researchers according to their impact on the research area and highlights the key aspects covered by leaders in this field.

## 2. Methods

To determine the most important topics and assess the academic impact of environmental valuation, we performed a bibliometric analysis considering publications in the Web of Science from 1987 to 2019. We assessed their productivity through their historical evolution and the distribution of papers by journal. The units of analysis were ordered by the citation and co-citation structure and the results gave insights into the organization and future trends on research in environmental valuation.

We performed a search for papers containing the term “environmental valuation” in the title, abstract or keywords. The search was conducted on the online literature search engine of the Web of Science electronic databases. On 17 December 2019 we obtained 661 results from the search engine covering the period 1987–2019, including articles, book chapters, proceedings papers and reviews of 1442 authors. Table 1 shows the protocol followed to perform the data collection and some key figures.

The dataset is analysed in the following section on R [16], a free software environment for statistical computing and graphics. The bibliometrix [17] package was used to compile most of the tables in this paper.

## 3. Results

### 3.1. Environmental Evaluation Publication History

**Figure 2:**
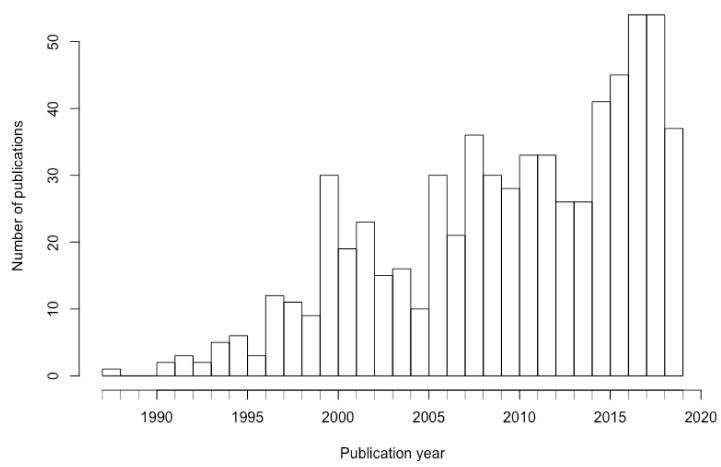
Distribution of Environmental valuation publications by year (1987–2019).

The number of publications per year is depicted in Figure 2. The first known paper on environmental valuation, published in 1987, was followed by a steady increase in number of environmental valuation-related publications over time.

Although the research was published in a wide range of journals, the 4 most popular were: Ecological Economics, Environmental & Resource Economics, Environmental Values, and Journal of Environmental Management– with nearly 30% of the studies (Table 2). Ecological Economics stands out as the most prolific source on this subject with 109 papers, which represents 16.5% of the total sample. Not surprisingly, the top Journals are particularly involved with environmental and ecological issues. The first 6 Journals are grouped into Environmental Sciences or Environmental Studies categories from the Journal Citation Reports of the Web of Science. When taking the impact factor into consideration, the top 6 Journals were ranked into the first quartile of their corresponding categories in 2018, while the rest of Journals are between the first and second quartile in most cases.

### 3.2. Leading Topics in Environmental Valuation Research

The most common keywords used by researchers include “environmental evaluation”, “willingness to pay”, and “ecosystem services” (Table 3). The keyword “environmental valuation” was used in 38% of the publications analyzed. The following keywords give useful insights into the evolution of the research topic and the methods developed and applied to value environmental goods and damage. The second most often used keyword is “willingness to pay”, which is commonly found in publications related to stated preference methods. The two abovementioned approaches to this group of environmental valuation methods occupy positions 4 (choice experiment) and 5 (contingent valuation). The choice experiment method also appears in the 7th position as “choice experiments”. The total of both alternatives (78) comes just after the “environmental valuation” keyword.

As the search procedure is automatic, the system differentiates “Choice experiment” from “Choice experiments”. In order to consider all the possible synonyms, we conducted a new experiment by searching for individual terms in the keywords (Table 3). For example, the word “choice” was used to collect all the papers with a keyword related to the choice experiment method. This provided similar expressions to those given in Table 3: Choice modeling, Choice modelling, Choice model, Choice experiment method, etc. The analysis showed that keywords related to the choice experiment method appeared in 165 papers, while other methods had a lower frequency (contingent valuation method, 69; hedonic price method, 18; travel cost method, 11).

**Figure 3:**
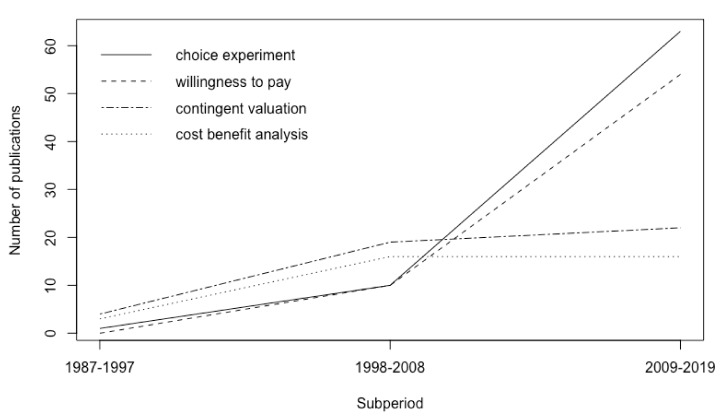
Evolution of the main keywords used by researchers in environmental valuation.

The relevance of choice experiments as a prominent keyword used by researchers has increased over time. We show the evolution of four keyword categories through 3 equally spaced subperiods: 1987–1997, 1998–2008 and 2009–2019 (Figure 3). The first two subperiods were dominated by keywords associated with contingent valuation methods (with labels “contingent valuation” and “contingent valuation method”) and cost-benefit analysis. However, a sudden change was found in the trend during the subperiod 2009–2019. During this time the choice experiments (with labels “choice experiment”, “choice experiments” and “choice experiment model”) dominated the researchers’ interest, closely followed by the willingness to pay keyword.

The popularity of the choice experiment method –over the contingent valuation method—was predicted by Adamowicz [14]: “The most significant advance in environmental valuation may be to move away from a focus on value and focus instead on choice behaviour and data that generate information on choices.” We can suggest several reasons to support the observed trend. First, the design of both methodologies makes the choice experiment method to extract more information than the contingent valuation method does. Results from contingent valuation are elicited by asking respondents for their willingness to pay (or willingness to accept). In a bidding game, the respondent is asked if he is willing to pay a specific amount of money. If the answer is yes, a higher amount is asked and, if the answer is no, a lower amount is proposed. The questionnaire is repeated until an initial yes changes to a no or vice versa. However, the choice experiment method uses attributes to define alternatives and information of the willingness to pay is obtained by observing the choices made by respondents [18]. As stated by Hoyos [15], the choice experiment method allows estimating the mean willingness to pay and also the marginal willingness to pay for the different attributes. Handling with more alternatives and attributes makes the application of the choice experiment more complex. However, its implementation has been facilitated by the development of statistical software. Furthermore, web-based surveys are becoming popular and easy to implement and the number of connected people to the internet keeps increasing, which limits biased sampling, then allowing presenting the choice set in a friendly manner [19]. An additional benefit from using the choice experiment method is related with the sensitivity to scope. This is one of the main concerns about the contingent valuation method, where the use of labels in the choice experiment may mitigate the lack of sensitivity to the scope [19].

### 3.3. The Most Influential Authors in Environmental Valuation

The most prominent authors in an area of research can be identified by citation analysis. Of the top 10 most influential publications on environmental valuation according to the number of citations, Boxall and Adamowicz [20] leads with 527 citations (Table 4). The authors use a latent class model to evaluate choice behaviour as a function of observable attributes of the choices and latent heterogeneity in the respondents’ characteristics. Although it has the highest number of citations, the paper by Lancsar and Louviere [21] received more cites on a yearly basis. The choice experiment model dominates the top ranked papers of Table 4 in which the authors introduce different environmental valuation examples to illustrate their proposals. Some of the top ranked papers are devoted either to the demonstration of case studies or to a review of the literature.

**Figure 4:**
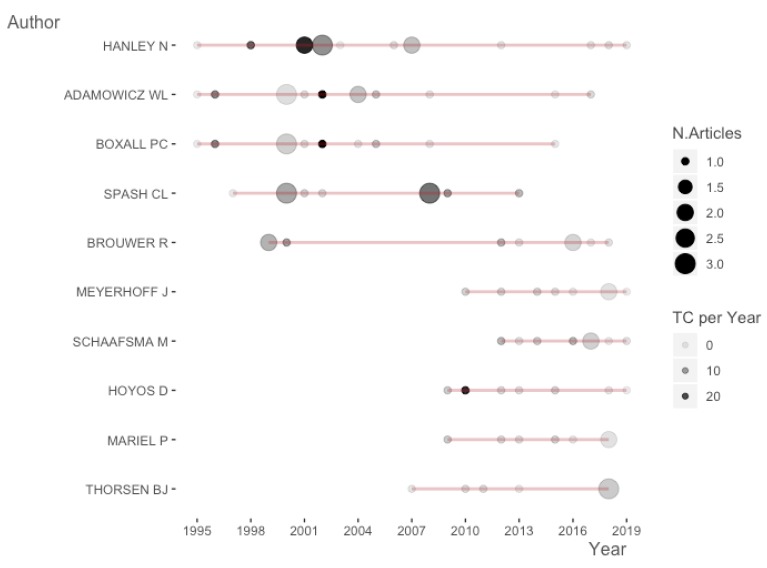
Relevance of authors according to their production and the number of citations.

We have analyzed the relevance of different authors in the topic according to the number of publications and the number of citations per year. Figure 4 gives one line to each author, where the extremes represent the year of the first (left circle) and last publication (right circle). Hanley was cited for the longest period, which was 25 years (1995–2019). The diameter of the circles varies in proportion to the number of papers published each year and the colour denotes the number of cites received. For example, the paper by Hanley et al. [22] has the highest number of citations per year (21.9) in the table. Although this is not the most cited paper according to the bibliographic analysis, it appears in the figure because Hanley is the most prolific researcher.

The figure distinguishes two groups of authors. The first incorporates those who have been publishing on the topic for roughly 20 years: Hanley, Adamowicz, Boxall, Spash and Brouwer. The other group contains those who published between 2007 and 2019: Meyerhoff, Schaafsma, Hoyos, Mariel and Thorsen.

**Figure 5:**
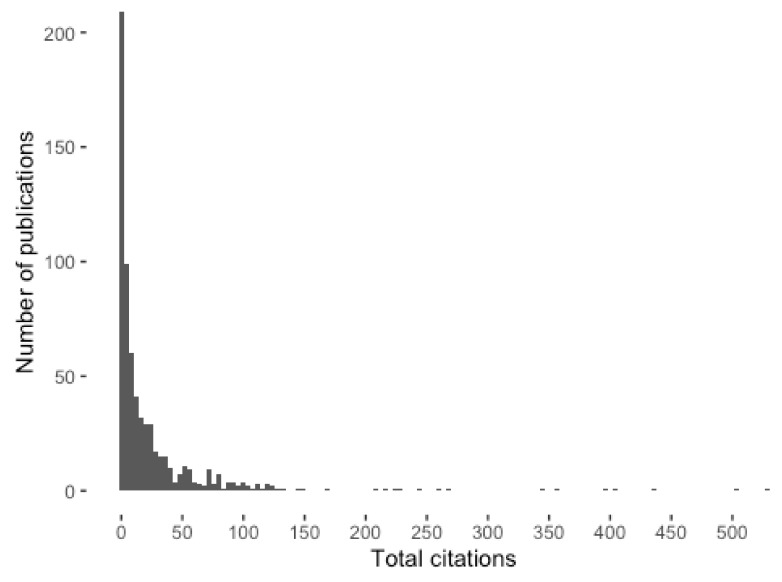
Distribution of citations per paper.

It should be noted that a few papers are responsible for a high percentage of the citations (Figure 5). gives the number of citations in descending order. Only 7 papers received more than 300 citations for the whole period analyzed, while 55.7% received 10 or fewer. This shows that only a few papers influenced this research topic during this period.

**Figure 6:**
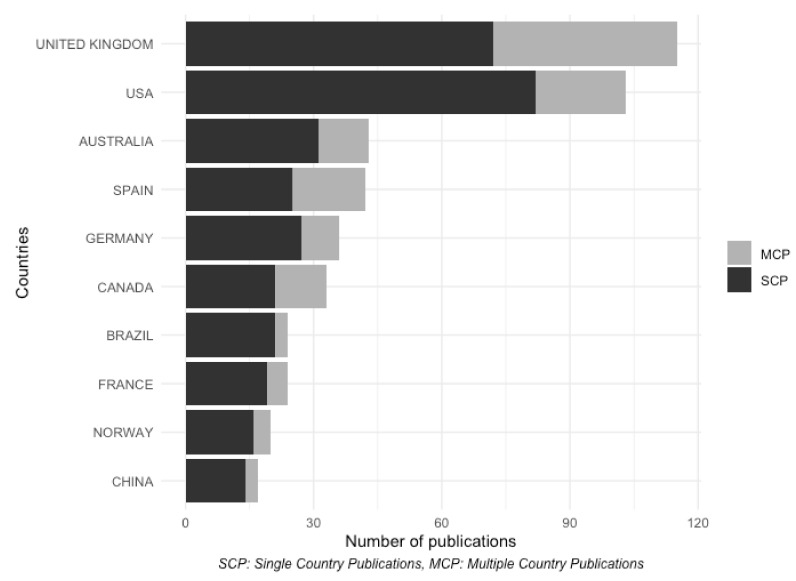
Most productive countries in environmental valuation.

Lastly, there is another interesting point related to the authors’ affiliation country; Figure 6 separates the papers whose authors’ affiliations are all located at the same country (Single Country Publications, SCP) and those with authors’ affiliations from different countries (Multiple Country Publications, MCP). The UK and the USA dominate the research on environmental valuation according to the number of papers published during the analyzed period. There are only 5 European countries in the top 10, while China is the only Asian representative. China is also in the last position in the top 10. Regarding the collaboration between authors from different countries, researchers from the UK and Spain are the most likely to collaborate in multinational publications, while Brazilian and Chinese affiliations produced the fewest publications with contributions from foreign authors.

### 3.4. Co-Citation Analysis

**Figure 7:**
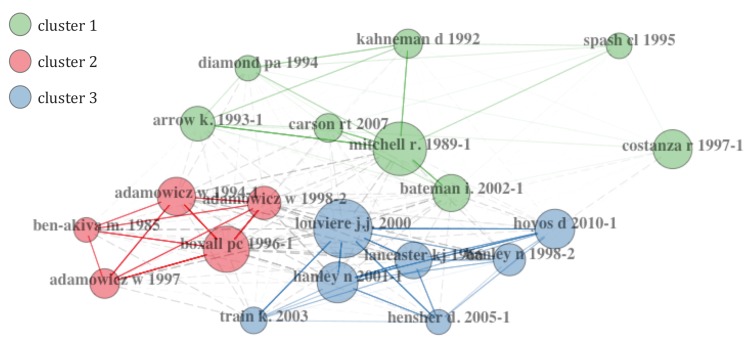
Co-citation network analysis.

This subsection begins with some comments about what productivity is in the field of research publication. Of course this is a wide field of debate, but some preliminaries must be established before proceeding with the co-citation analysis. According to [29], there are several measures to account for productivity. The most basic bibliometric measure is the number of papers published, which provides the raw data for all citation analysis. Another measure is the number of citations, which determines the recognition and influence of a paper. Then we can distinguish between citations received from papers published in Journals indexed in WoS, or citations received for other Journals not considered in WoS. As stated by [29], a measure of association between highly cited papers is used to form clusters: “That measure is the number of times pairs of papers have been co-cited, that is, the number of later papers that have cited both of them”. Hence, co-citation implies that two papers are cited in a third paper and assumes that both papers are related. We have performed a co-citation analysis by differentiating 3 main clusters in different colours (Figure 7). The references of cluster 1 are represented by the book by Mitchell and Carson [30], in which the authors describe the contingent valuation method and claim that “the contingent valuation (CV) method offers the most promising approach for determining public willingness to pay for many public goods”. However, the positivist perspective in Mitchell and Carson [30] is contested by other prominent works in the same group. The report in Arrow et al. [31] indicate several drawbacks to the contingent valuation method and gives some guidelines to be used if the proposal is to produce useful information for natural resource damage assessment. The research in Kahneman and Knetsch [32] reports the most serious shortcoming of the CV method. According to these authors: “the assessed value of a public good is demonstrably arbitrary, because willingness to pay for the same good can vary over a wide range depending on whether the good is assessed on its own or embedded as part of a more inclusive package”. There is a more recent relevant book in this group, Bateman et al. [33], which gives a general approach to stated preferences techniques with application to different non-market goods and services.

The cluster 2 (in red) elicited from the co-citation analysis is led by the paper by Boxall et al. [26], “A comparison of stated preference methods for environmental valuation”. This paper introduces an empirical comparison of the contingent valuation method and choice experiments. Most papers in this group follow the approach in Boxall et al. [26]. For example, Adamowicz et al. [34] examine the choice experiment as “an extension or variant of contingent valuation”. The paper in Adamowicz et al. [35] had earlier compared a stated preference model and a revealed preference model for recreational site choice. The earliest work in the group is the book by Ben-Akiva et al. [36], which analyzes the discrete choice method from a more general perspective.

And lastly, the cluster 3 covers different references related to choice modelling approaches but with a different approach to the publications in the second group. Again, a single book is the leader in number of cites: Louviere et al. [37]. Interestingly, this book is not the only reference which gives a survey of choice modelling. The paper by Hoyos [15] provides a review of the state of the art of environmental valuation with discrete choice experiments; Hanley et al. [22] examine the choice modelling approach to environmental valuation. The authors state that this methodology “can be considered as an alternative to more familiar valuation techniques based on stated preferences such as the contingent valuation method”; Hanley et al. [23] also outline choice experiments and analyze its roots in Lancaster’s characteristics theory of value; while the paper by Lancaster [38] is another relevant work in this group.

## 4. Discussion

Environmental valuation is intrinsically difficult because realistic environmental valuation situations are rarely observed, and singularities in environmental assets impede a uniform treatment of those values outlined by the Total Economic Value. Notwithstanding the difficulties, a plethora of papers have been published during the last decades.

As a result of this research it can be concluded that revealed preferences methodologies are surpassed by works focused on stated preference methods for the analyzed period as a whole. The research discloses the relevance of stated preference methods over revealed preferences methods, with a clear dominance of choice experiment over any other environmental valuation method, as predicted by Adamowicz [14]. The complexity of the choice experiment method has resulted in new challenges and research lines for academics. Choosing and implementing experimental designs, interpreting standard and more advanced random utility models, and estimating measures of willingness-to-pay are some of the issues covered by researchers [39].

Differences on the environmental valuation have been also revealed by the co-citation analysis, which reports different clusters by considering the methods used in the environmental valuation process. Despite its past influence, none of the travel cost and hedonic price methods is in the 10 most popular methods of environmental valuation, according to the keywords in the dataset used. In addition, the leading Journals in the publication of environmental valuation papers are ranked in prominent positions by WoS in their corresponding categories. The paper also distinguish two groups of authors according to the time they have published on the topic. The first group initiates the growth of the area in the mid-1990s, while the second group concentrates its impact mainly from 2010.

The abovementioned differences in the use of the environmental valuation methods do not imply that one method is unequivocally better or worse than another since its appropriateness depends on a particular situation. In other words, no single method is suitable in all valuation scenarios. Rather, the choice of the valuation method is context-specific. Revealed preference methods can be prioritized when budget and time are constrained. Stated preference methods require a complex questionnaire development and data analysis, which translates into an additional need of resources (both money and time). Conversely, revealed preference methods can only capture use values, while stated preference methods can estimate both use and non-use values. In addition, using multiple methodologies can be appropriate in some situations. For example, the combination of revealed and stated preference methods can improve benefit estimation of a single component [35]. This approach can be useful when a revealed preference method is utilized as the main valuation instrument, but some environmental values are more accurately estimated by using another method and the result is aggregated. In this case, the researcher must be careful to avoid double counting if the components of value captured by the different methods overlap [40].

## 5. Conclusions

From the evolution of environmental valuation publications in the last 30 years, we can assert that the discipline has been consolidated. Papers related to choice experiments have dominated academic production in the last decade. In the current stage of environmental valuation researchers will have to cope with new challenges and emerging trends. As in other research areas, the increasing ability to collect enormous amounts of data facilitates the creation of the available massive databases, which can be used to take environmental valuation methodologies to the next stage in their evolution by incorporating machine learning techniques in the valuation process. However, this evolution should not be restricted to new applications of the well-known valuation methods only. Researchers must develop new approaches to deal with new elements in the valuation process. We expect that climate change, as one of the defining challenges of the 21st century, will attract most attention from researchers to propose new approaches in environmental valuation [41,42]. As knowledge and perception are subjective, the intangible aspects must be explicitly considered in the new valuation methods [13]. In this regard, we may conclude that the future path of environmental valuation is not necessarily related to new methodologies, but to the inheritance and assimilation of consolidated techniques commonly used in other scientific areas.

## Figures and Tables

**Table 1:** Procedure for the data collection and key figures.

Database	Web of Science
Period	All years to 2019
Search date	17 December 2019
Search terms	“Environmental valuation”
Information retrieved	Title, Keywords, Authors, Journal, Year, Impact factor, Number of citations, Document type
Number of documents	661
Sources (Journals, books, etc.)	289
Authors	1442
Authors of single-authored documents	134
Authors of multi-authored documents	1308
Average citations per document	25.79

**Table 2:** Most relevant journals that have published the greatest number of environmental valuation papers.

Position	Journal	Number of Papers	% of Papers
1	Ecological Economics	109	16.5%
2	Environmental & Resource Economics	36	5.4%
3	Environmental Values	23	3.5%
4	Journal of Environmental Management	19	2.9%
5	Ecosystem Services	11	1.7%
6	Land Use Policy	11	1.7%
7	Environment and Planning C-Government and Policy	10	1.5%
8	Journal of Environmental Planning and Management	10	1.5%
9	Land Economics	10	1.5%
10	American Journal of Agricultural Economics	8	1.2%

**Table 3:** The 10 most used keywords by number of publications related with environmental valuation.

Position	Keyword	Number of Papers
1	Environmental valuation	251
2	Willingness to pay	64
3	Ecosystem services	51
4	Choice experiment	48
5	Contingent valuation	45
6	Cost benefit analysis	35
7	Choice experiments	30
8	Non market valuation	24
9	Valuation	24
10	Stated preference	23

**Table 4:** The 10 most frequently cited papers on environmental valuation.

#	Title	Authors	Journal	Year	Total Citations	Citations by Year
1	Understanding heterogeneous preferences in random utility models: a latent class approach	Boxall and Adamowicz [20]	Environ. Resour. Econ.	2002	527	27.7
2	Conducting discrete choice experiments to inform healthcare decision making	Lancsar and Louviere [21]	Pharmaecon	2008	505	38.8
3	Choice modelling approaches: a superior alternative for environmental valuation?	Hanley et al. [22]	J. Econ. Surv.	2001	437	21.9
4	Using choice experiments to value the environment	Hanley et al. [23]	Environ. Resour. Econ.	1998	406	17.7
5	Valuing nature: lessons learned and future research directions	Turner et al. [24]	Ecol. Econ.	2003	395	21.9
6	Weak comparability of values as a foundation for ecological economics	Martinez-Alier et al. [25]	Ecol. Econ.	1998	356	15.5
7	A comparison of stated preference methods for environmental valuation	Boxall et al. [26]	Ecol. Econ.	1996	346	13.8
8	The state of the art of environmental valuation with discrete choice experiments	Hoyos [15]	Ecol. Econ.	2010	267	24.3
9	Designs with a priori information for nonmarket valuation with choice experiments: A Monte Carlo study	Ferrini and Scarpa [27]	J. Environ. Econ. Manag.	2007	260	18.6
10	Perceptions versus objective measures of environmental quality in combined revealed and stated preference models of environmental valuation	Adamowicz et al. [28]	J. Environ. Econ. Manag.	1997	245	10.2

## Abbreviations

The following abbreviations are used in this manuscript:

MCP	Multiple Country Publications
SCP	Single Country Publications
TEV	Total Economic Value
WoS	Web of Science
